# Formulation and Characterization of Food Hydrogels: Gelation Mechanisms, Dehydration Pathways, and Effects of Embedded Plant Cells

**DOI:** 10.3390/gels12080659

**Published:** 2026-07-23

**Authors:** Rocco Carcione, Valentina Mastrobuono, Riccardo Pagliarello, Elisabetta Bennici, Alessia Cemmi, Silvia Massa

**Affiliations:** 1 ENEA, Nuclear Department, Research Center Casaccia, Via Anguillarese 301, 00123 Roma, Italy; 2 ENEA, Department for Sustainability, Sustainable Agri-Food Systems Division, Agriculture 4.0 Laboratory, Via Anguillarese 301, 00123 Roma, Italy; v.mastrobuono96@gmail.com (V.M.); riccardopagliarello@gmail.com (R.P.); elisabetta.bennici@enea.it (E.B.)

**Keywords:** food-grade hydrogel, agri-food by-products, gelation mechanisms, time-resolved FTIR/ATR, plant cell cultures, water retention

## Abstract

The development of sustainable food systems increasingly relies on the valorization of agri-food by-products and circular economy strategies. In this context, food-grade hydrogels represent promising matrices for incorporating biological components and bioactive compounds. This study focuses on the preparation and characterization of four hydrogels formulated with natural biopolymers, such as pectin, an extract obtained from spray-dried by-products generated during the processing of strawberry and blueberry fruit preparations, with potential applications in innovative and sustainable food formulations. A specific formulation was produced by including plant cells. UV–visible spectra, pH, and moisture content of the hydrogels were evaluated. Their morphological, chemical, and hydrodynamic properties were investigated using micro-Raman and FTIR/ATR spectroscopy along with swelling degree and dehydration behavior over time. The analysis clarified gelation mechanisms, confirming the key role of calcium chloride in matrix stabilization and the influence of plant cells on water retention. The developed food-grade hydrogels demonstrated physicochemical stability, even after 20 days of storage at 4 °C. The incorporation of plant cells modulated the hydrogel’s hygroscopic behavior, maintaining the fundamental gelation pathways. These results highlight the potential of these hydrogels as sustainable matrices for incorporating biological components and support their application in innovative food formulations based on the valorization of by-products.

## 1. Introduction

The sustainable management of food waste and the valorization of agri-food by-products represent major challenges and opportunities for the agri-food industry. While the accumulation of food waste leads to significant environmental impacts and the loss of valuable resources, agri-food by-products generated during food processing constitute secondary raw materials that can be efficiently recovered within a circular bioeconomy framework. Numerous studies have demonstrated that these by-products can be transformed into value-added food ingredients, contributing to the development of innovative and sustainable food products, improving their nutritional profile, enhancing resource efficiency, and reducing the amount of waste requiring disposal [1].

Among the most interesting systems are gels, and in particular, food-based hydrogels, which constitute three-dimensional matrices capable of retaining water and incorporating various components within their structure. Thanks to their physicochemical and hygroscopic properties, hydrogels can be used as versatile matrices for incorporating bioactive compounds, plant extracts, powders derived from agri-food by-products, or even plant cells, while maintaining structural stability at the molecular level [2,3]. This ability makes these systems particularly promising tools for the development of new sustainable food materials.

One of the most relevant biopolymers used for hydrogel formation is pectin, owing to its natural origin, its widespread presence in the human diet, and its functional properties. Pectin is a structural polysaccharide present in the primary cell wall and middle lamella of higher plants and is widely used in the food industry for its gelling properties [4]. It is mainly extracted from plant by-products such as citrus and apple peels, thus representing a significant example of the valorization of agri-food residues. In addition to its technological role, pectin is also a soluble dietary fiber associated with several health benefits, including cholesterol reduction and the ability to bind heavy metals in the gastrointestinal tract [4].

Based on their chemical composition, pectins can be classified according to their degree of methoxylation into high-methoxyl (HM) and low-methoxyl (LM) pectins, which exhibit different gelation mechanisms [5]. In particular, LM pectins form gels in the presence of divalent cations, such as Ca^2+^, through the so-called “egg-box” model, in which calcium ions stabilize the three-dimensional network of the polymer matrix, allowing for the retention of water and solutes [6,7]. The gelation process can be modulated through various parameters, including pH, calcium concentration, temperature, and the presence of sugars or salts, allowing control over the hydrogel’s mechanical and structural properties [8,9]. In particular, pectin gelation mechanisms fundamentally dictate the three-dimensional architecture and functional performance of food hydrogels. The current literature establishes a general mechanism centered on the cross-linking of galacturonan chains [7,10,11,12,13]. In this framework, the presence of calcium ions is crucial for activating the ‘egg-box’ model through stereospecific binding to carboxylic residues, thereby stabilizing the network at a molecular level [5,6,10]. This precise physicochemical environment provides an optimal template for the incorporation of living plant cells into the hydrogel matrix. This strategy effectively mimics the native extracellular matrix of plant tissues, significantly enhancing the hygroscopic profile, water retention, and predictability of the final system at the molecular structural level [2,14].

In this context, the use of extracts obtained from agri-food by-products or fruit juices may represent an interesting strategy for formulating functional hydrogels. These ingredients, in addition to contributing to the regulation of pH and pectin gelation conditions, can enrich the matrix with bioactive compounds and molecules of nutritional interest, promoting the valorization of by-products and the development of innovative and sustainable food materials.

Emerging technologies such as 3D food printing have increased interest in the use of ingredients derived from processing residues. Although this technology is primarily known for its ability to produce customized food shapes, it relies on the availability of materials with suitable structural properties, and hydrocolloid-based systems have been extensively studied in this context in the scientific literature [15,16]. Furthermore, food-processing by-products have been reported to contribute to the physicochemical properties of food formulations, particularly in terms of chemical compositional stability [17]. This valorization strategy aligns with the concept of “from waste to wealth,” which promotes the transformation of food-processing residues into resources for the development of more sustainable food systems [18]. Furthermore, the incorporation of living plant cells into food-grade hydrogel matrices aligns with the emerging research field of plant cell agriculture [19,20]. This field investigates alternative strategies for the production of plant-derived biomass under controlled conditions, aiming to complement conventional agricultural systems through more standardized and reproducible approaches.

This study focuses on the preparation and characterization of food hydrogels formulated with natural biopolymers and extracts derived from agri-food by-products. The chemical and hydrodynamic properties of the hydrogels; the UV–visible spectra, pH and moisture content of individual hydrogels; and water retention, swelling, and dehydration were analyzed to understand gelation mechanisms and the role of calcium in matrix stabilization. In particular, the core innovation of this work lies in the comprehensive rationalization of the matrix properties achieved by systematically combining all these analytical techniques. Through this integrated approach, this work provides a deep, multi-parametric evaluation of hydrogels integrating multiple distinct by-products. Most notably, we investigate the active role of living plant cells in modulating hydrogels’ physicochemical, kinetic, and hygroscopic behavior. By deploying systematic, time-resolved spectroscopic studies, we establish a direct link between dehydration mechanisms, compositional chemical evolution, and long-term chemical stability. Consequently, this work moves beyond simple ingredient blending, offering a novel, rationalized perspective on how living components and diverse by-products cooperatively dictate the physicochemical integrity of food-based hydrogels.

## 2. Results and Discussion

### 2.1. Hydrogels’ Formulation and Visual Appearance

The main focus of the present study concerns the chemical and hydrodynamic characterization of four hydrogels, with particular attention paid to the gelation mechanisms and the role of calcium chloride in stabilizing the pectin matrix.

The formulation process of the four food hydrogels followed an iterative trial-and-error strategy. The ingredients were selected based on the technological and nutritional properties of the individual components, including value-added agri-food by-products, apple juice, pectin, and calcium chloride. The formulations were subsequently optimized by progressively adjusting the concentrations of the different components. The resulting formulations, developed to investigate the interactions among the ingredients during the structuring process, were empirically evaluated through visual inspection and manual extrusion using a syringe (2 mL BD Emerald 1 mm diameter), resulting in satisfactorily structured filaments, characterized by a uniform structure that maintained its shape without collapsing after extrusion.

Spray-dried strawberry and blueberry by-product powders (1 g) were dispersed in 100 mL of aqueous citric acid solutions (0.5 or 5% *w*:*v*) to obtain the corresponding extracts. These extracts were subsequently used as the liquid phase in the hydrogel formulation. For clarity and comparability across formulations, only the mass fraction of the components (% wt) is reported in the composition table (Table 1), while water is not included as a separate component.

Citric acid (E330), a food-grade organic acid commonly used as an acidity regulator, was selected due to its ability to solubilize polar phenolic compounds. In this context, its extraction efficiency is comparable, although milder, to that of conventional polar organic solvents such as methanol, making it suitable for food applications.

Beyond its role as an extraction solvent, citric acid also modulates the pH within the hydrogel formulation, which contributes to the physicochemical stability of the matrix. This chemical stabilization helps maintain the molecular integrity of the system and hygroscopic aspects of the network.

In hydrogel 1, the inclusion of apple fiber powder and high-oleic oil improved the molecular structural stability and nutritional profile by enhancing fiber and lipid. Honey was incorporated to further stabilize the matrix, leveraging its natural crystallization tendency. The inclusion of locust bean gum in hydrogel 2 significantly enhanced the hydrogel’s physicochemical stability and molecular network cohesion, while ingredients such as concentrated apple juice and banana powder enriched the formulation with bioactive compounds, including proteins and phenolic compounds. Hydrogel 3, specifically designed for the inclusion of viable plant cells, presented the challenge of maintaining the vitality of the cells within a gelled matrix. Finally, hydrogel 4 was adjusted to obtain a gummy texture. Increasing honey allowed for gelation without the need for calcium chloride.

**Figure 1:**
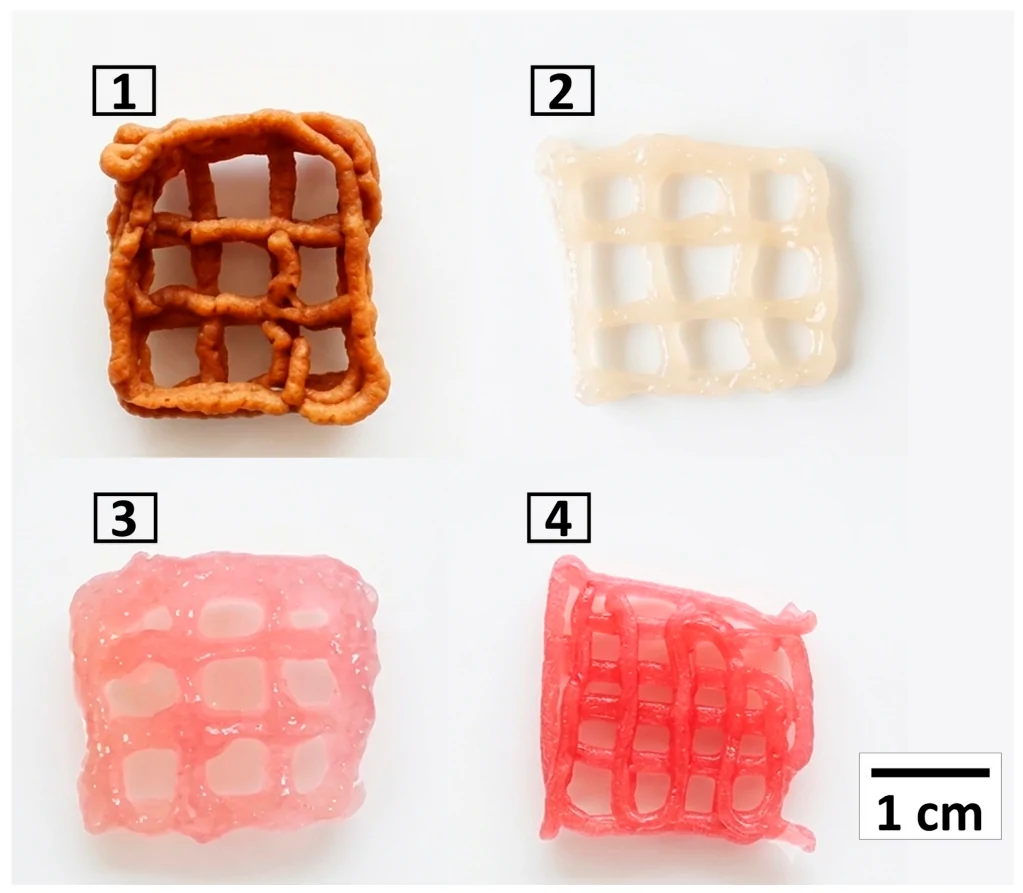
Macroscopic appearance of the extruded hydrogels (1, 2, 3, and 4) obtained after manual extrusion using a syringe (2 mL BD Emerald, 1 mm nozzle diameter).

The visual appearance of the deposited hydrogel samples (1, 2, 3, and 4) is shown in Figure 1.

### 2.2. Evaluation of Effects of UV–Visible Spectra, pH and Moisture Content on the Hydrogels

The four final recipes were evaluated through UV-Vis spectroscopy of their hydroalcoholic extracts, alongside monitoring of moisture content and pH at 4 °C over time (0–96 h) as indicators of their physicochemical stability. Furthermore, the observed consistency was considered potentially advantageous for maintaining a heterogeneous morphology after deposition, as it can reduce the tendency of the material to collapse compared to more fluid formulations.

- Hydrogel 1

**Figure 2:**
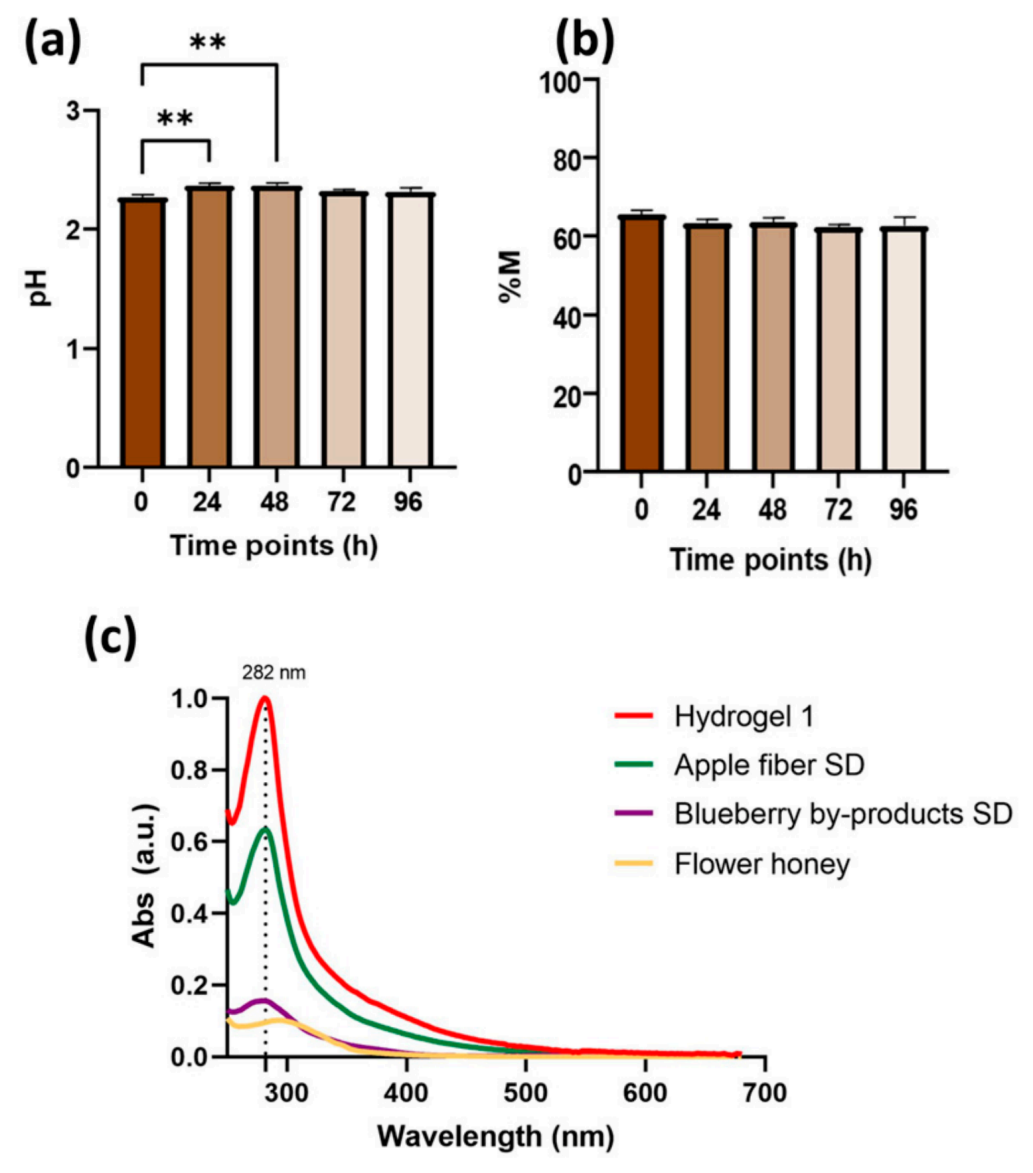
Study of the variation in (**a**) pH and (**b**) humidity over time (0–96 h) of hydrogel 1 using Tukey’s multiple comparison test (Ti: 0 h; Tf: 96 h) for statistical significance. (**c**) UV-Vis spectra of MeOH:H_2_O 70:30 *v*:*v* extracts of hydrogel 1 and its ingredients (SD: spray-dried; Abs: absorbance) at T0. Real (calculated) O.D. values are reported in the table, and arbitrary units (a.u.) in the spectra. Error bars represent standard deviation (SD). Asterisks indicate statistically significant differences (** *p*-value ≤ 0.01).

The data indicate an increase in pH from 0 to 48 h (Figure 2a). This basification may be attributable to the progressive degradation of citric acid over time, as well as the acid hydrolysis of polysaccharides present in apple fiber [21,22]. However, the overall pH variation was limited to approximately 0.1 pH units, indicating good pH stability throughout the storage period. The total moisture content of the freshly deposited sample was 65.50%, with only minimal variations observed during the 96 h storage period at 4 °C (Figure 2b). The data obtained by the analysis of UV-Vis spectra are reported in Figure 2c and Table 2. A prominent absorption maximum at 282 nm was consistently reflected in the spectra of the individual raw components, specifically the spray-dried apple fiber, confirming that the hydrogel can successfully retain the bioactive profile of its ingredients. This specific absorption peak is characteristic of flavonoids, likely present in spray-dried apple fiber [23]. The high absorbance intensity of the apple fiber at this wavelength suggests it is a primary contributor to the polyphenolic concentration of the hydrogel.

- Hydrogel 2

**Figure 3:**
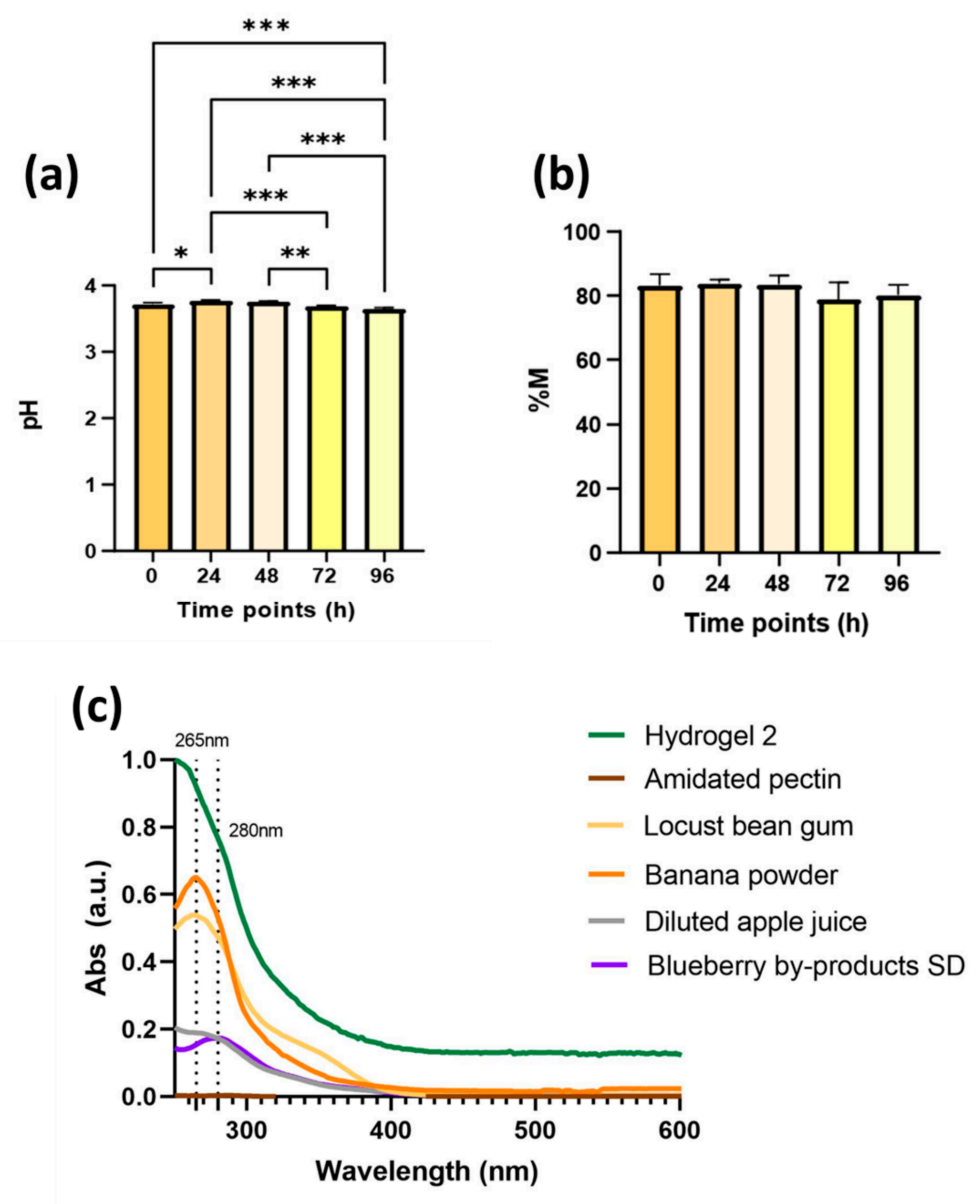
Study of the variation in (**a**) pH and (**b**) humidity over time (0–96 h) of hydrogel 2 at 4 °C using Tukey’s multiple comparison test (Ti: 0 h; Tf: 96 h). (**c**) UV-Vis spectrum of MeOH:H_2_O 70:30 *v*:*v* extract of hydrogel 2 in relation to that of its components (SD: spray-dried; Abs: absorbance) at T0. Real (calculated) O.D. values are reported in the table, and arbitrary units (a.u.) in the spectra. Error bars represent standard deviation (SD). Asterisks indicate statistically significant differences (* *p*-value ≤ 0.05; ** *p*-value ≤ 0.01 and *** *p*-value ≤ 0.001).

As shown in Figure 3a, the most pronounced pH variations were observed within the first 24 h post-preparation, characterized by a slight acidification trend. However, these fluctuations remained minimal, with changes confined to the first or second decimal place (e.g., pH 3.757 at 24 h compared to pH 3.633 at 72 h), indicating good stability in the pH profile of the hydrogel at 4 °C (Figure 3a). These minimal variations suggest an intrinsic buffering capacity of the formulation and the absence of significant chemical degradation pathways. This behavior is consistent with moisture stability (Figure 3b), collectively confirming its physicochemical reliability under the defined short-term storage conditions. The UV-Vis spectrum of the MeOH:H_2_O (70:30 *v*:*v*) extract (Figure 3c) exhibits a distinct absorption peak at 265 nm, consistent with characteristic UV-Vis signatures of phenolic acids, as reported for locust bean gum [24] and banana powder [25], both recognized as sources of this compound. The data obtained by the analysis of UV-Vis spectra are reported in Table 3.

- Hydrogel 3

**Figure 4:**
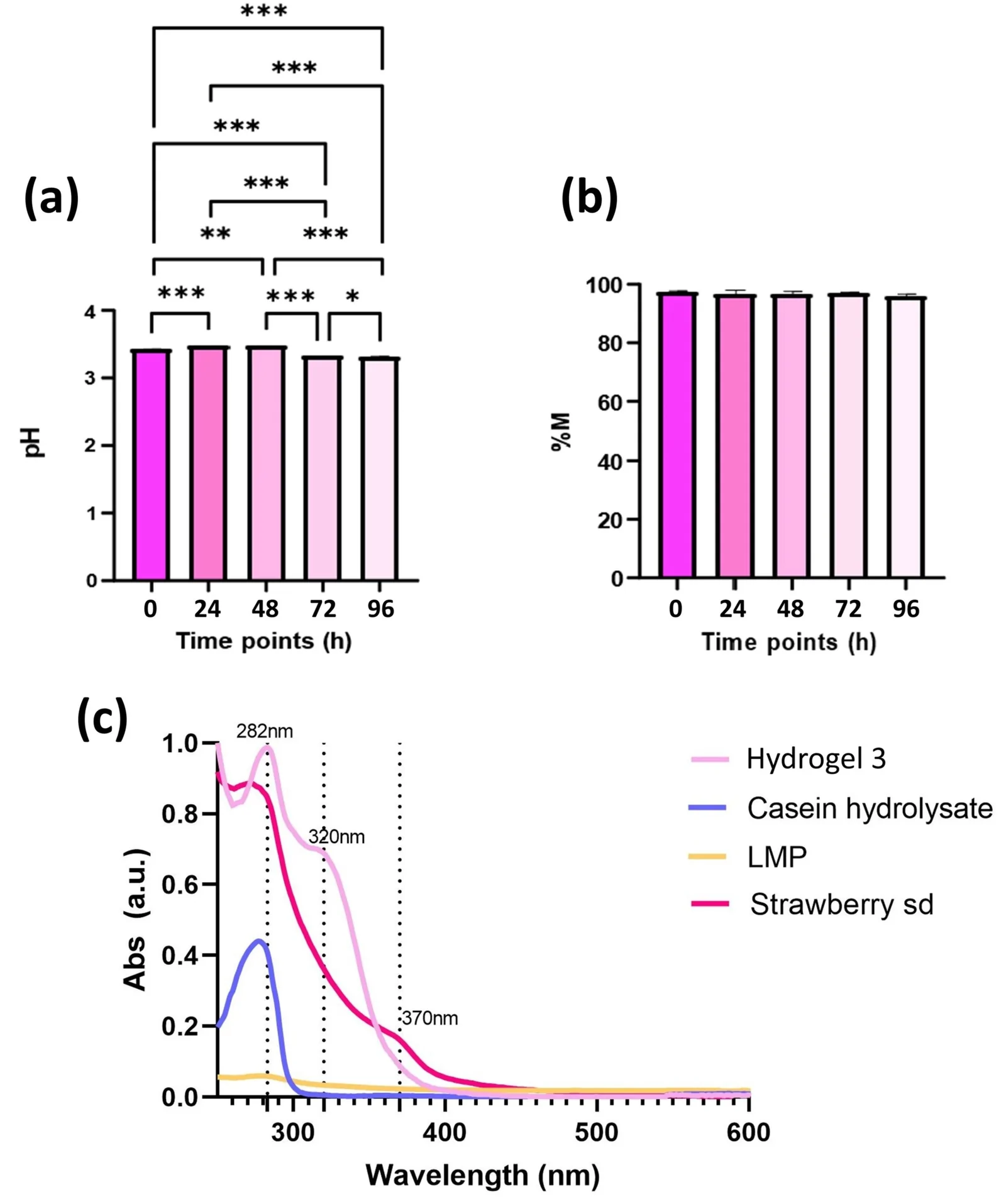
Study of the variation in (**a**) pH and (**b**) percentual humidity over time (0–96 h) of hydrogel 3 at 4 °C using Tukey’s multiple comparison test for statistical significance (Ti: 0 h; Tf: 96 h). (**c**) UV-Vis spectrum of MeOH:H_2_O 70:30 *v*:*v* extract of hydrogel 3 in relation with that of its components (SD: spray-dried; Abs: absorbance) at T0. Real (calculated) O.D. values are reported in the table, and arbitrary units (a.u.) in the spectra. Error bars represent standard deviation (SD). Asterisks indicate statistically significant differences (* *p*-value ≤ 0.05; ** *p*-value ≤ 0.01 and *** *p*-value ≤ 0.001).

As reported in Figure 4a, the pH of hydrogel 3 remains stable over time, although slight variations are observed within the first 24 h post-preparation. From 0 to 48 h, a gradual increase in pH is observed, followed by slight acidification, which may be influenced over time by the presence of calcium chloride in the hydrogel, as demonstrated in a study by Suriati et al. [26], where calcium chloride caused an increase in acidity during storage. This pH variation, however, remained within the first or second decimal place, highlighting the overall constancy of this parameter. This behavior is also reflected in complete moisture stability (Figure 4b). The UV-Vis spectrum of hydrogel 3 reveals a peak of absorbance at 278 nm, which is consistent with the presence of phenolic compounds derived from strawberry by-products [27]. Casein hydrolysate contributes to the overall absorbance at 280 nm. This observation agrees with the characteristic absorption of most proteins at this wavelength, primarily due to their tryptophan content [28]. The data obtained by the analysis of UV-Vis spectra are reported in Table 4.

- Hydrogel 4

**Figure 5:**
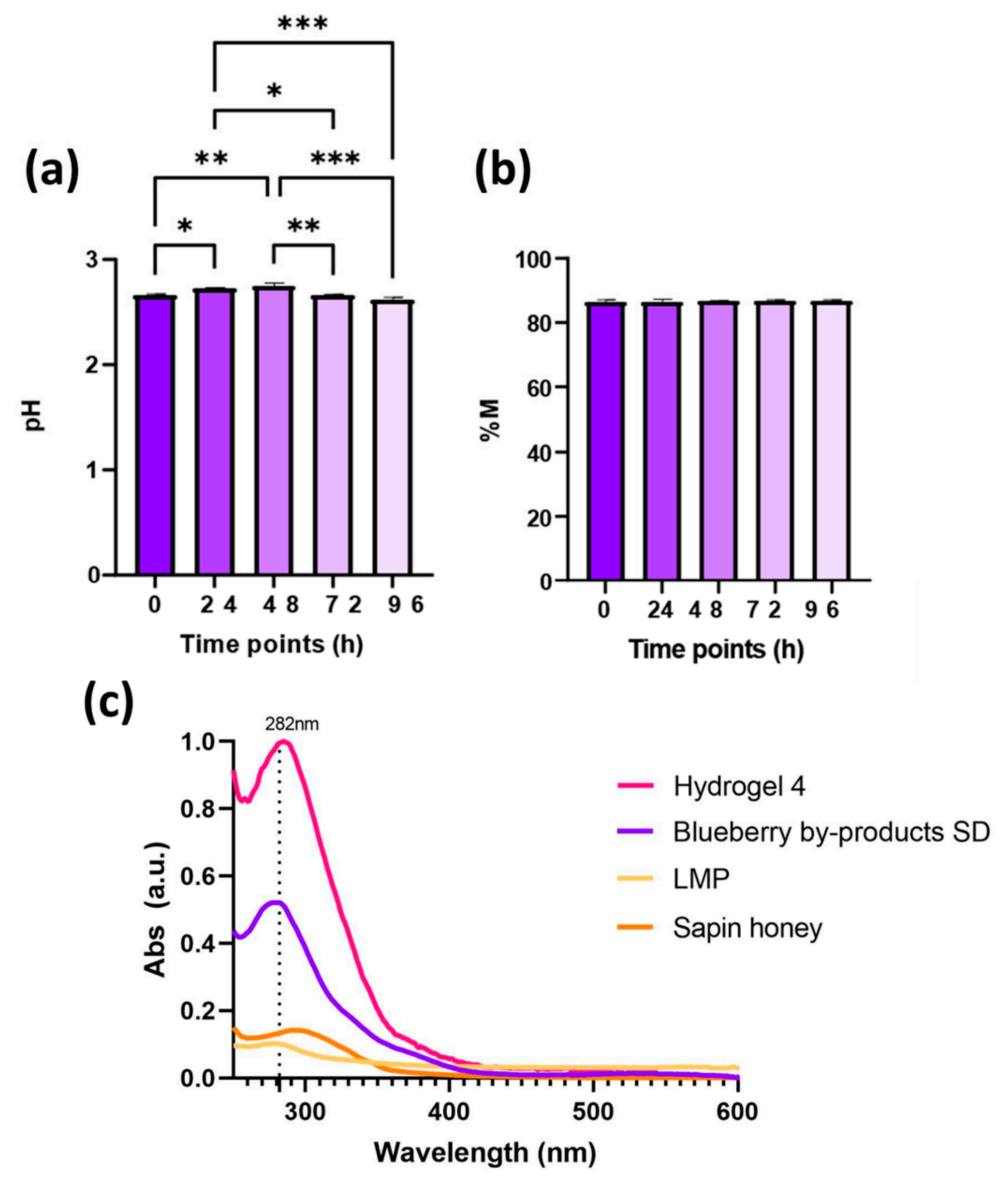
Study of the variation in (**a**) pH and (**b**) percentual humidity over time (0–96 h) of hydrogel 4 at 4 °C using Tukey’s multiple comparison test for statistical significance (Ti: 0 h; Tf: 96 h). (**c**) UV-Vis spectrum of MeOH:H_2_O 70:30 *v*:*v* extract of hydrogel 4 in relation to that of its components (SD: spray-dried; Abs: absorbance) at T0. Real (calculated) O.D. values are reported in the table, and arbitrary units (a.u.) in the spectra. Error bars represent standard deviation (SD). Asterisks indicate statistically significant differences (* *p*-value ≤ 0.05; ** *p*-value ≤ 0.01 and *** *p*-value ≤ 0.001

Slight acidification of pH was observed primarily between 24 and 96 h, and between 48 and 96 h (Figure 5a). However, these variations remain within the first or second decimal value, indicating stability of this parameter in hydrogel 4. Moisture content values ranged from 86,39% at 0 h to 86,86% at 96 h, showing only minimal fluctuation throughout the study period (Figure 5b). The UV-Vis spectrum shows an absorbance peak at 282 nm, attributable to flavonoids from blueberry (SD) and vanillin (fir honey) [29] (Figure 5c). The data obtained by the analysis of UV-Vis spectra are reported in Table 5.

### 2.3. Evaluation of P. frutescens Cell Suspension Culture’s Compatibility with Value-Added By-Product Extracts and Apple Juice, and Their Embedding into Hydrogel 3

*Perilla frutescens* suspension culture was evaluated based on its viability in either 1% strawberry- or blueberry-processing by-product extracts in 0,5% citric acid, or in clear apple juice concentrate diluted 5% in water (1 × 10^6^ cells/mL), as an index of compatibility of these matrices with plant cells compared to standard culture medium (2,4-D 0.5 mg/L and BAP 1 mg/L) as a control. Apple juice, due to the high °Brix value, required dilution to ensure a compatible osmolarity with plant cells and was therefore diluted (4.91:95.09, *v*/*v*) to achieve an osmolarity of 185 mOsm/L, comparable to the standard culture medium (211 mOsm/L), and thus suitable for *P. frutescens* cell viability.

**Figure 6:**
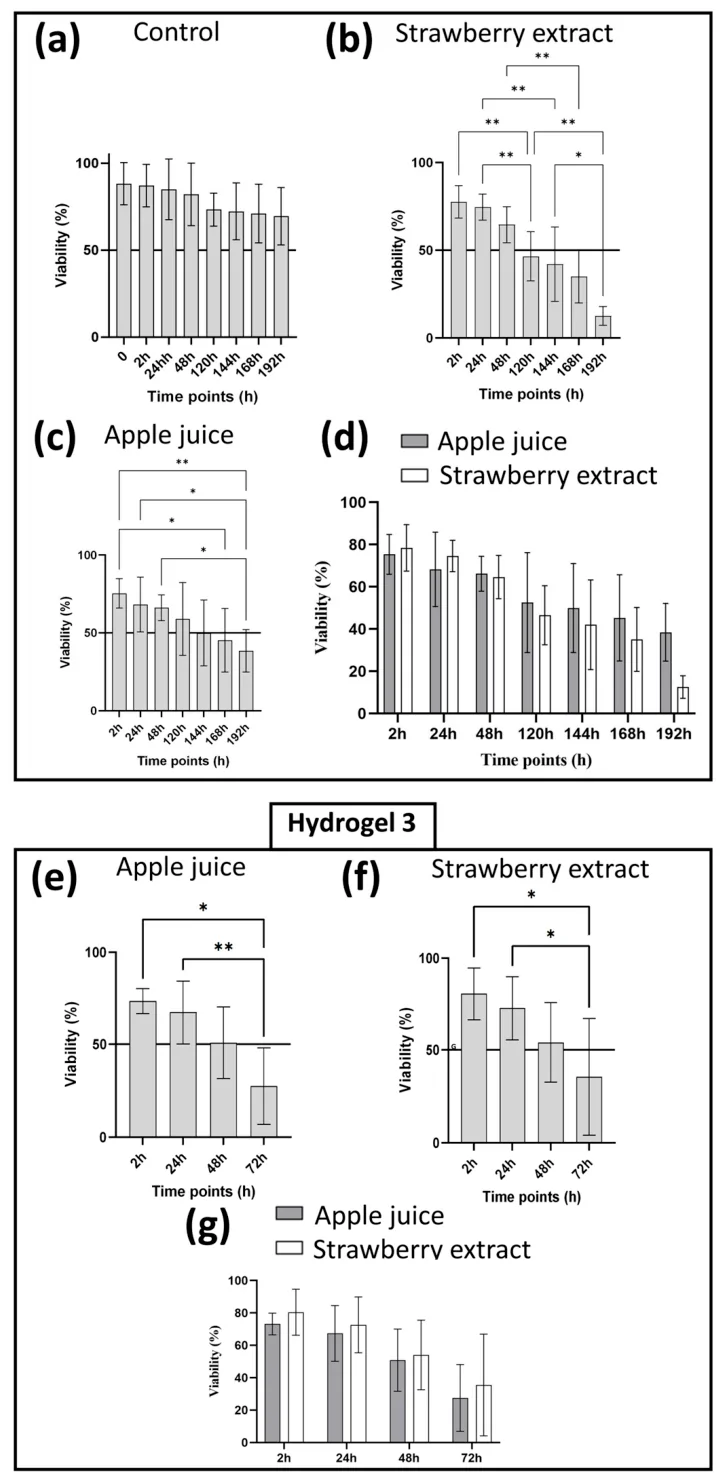
Viability of *P. frutescens* cells resuspended (**a**) in standard media as a control, (**b**) in 1% strawberry by-product extract in 0,5% citric acid or (**c**) in clear apple juice concentrate (diluted with double-distilled water) from T1 (2 h) to T8 (192 h, day 8). (**d**) Comparative analysis of cell viability between strawberry by-product extract (**b**) and diluted apple juice. (**c**,**e**) Viability of *P. frutescens* cells in hydrogel 3 with apple juice and (**f**) in hydrogel 3 with strawberry extract. (**g**) Comparative analysis of cell viability between the two hydrogel 3 formulations. (**e**,**f**) Data are reported as the mean ± standard deviation (SD) from two independent experiments. The analysis of variance was performed by one-way ANOVA with multiple comparisons and Tukey’s post hoc test. Asterisks indicate statistically significant differences (* *p*-value ≤ 0.05; ** *p*-value ≤ 0.01).

Viability was assessed for 192 h (8 days), maintaining samples at 4 °C, and the results are reported in Figure 6a–c as the mean of two experiments.

The viability of cells resuspended in blueberry by-product extract turned out to decrease to 0% between 2 h and 24 h; therefore, this extract was not further considered. This marked reduction in viability may be related to the physicochemical characteristics of the matrix, including acidity and the composition of the blueberry extract, which may have generated unfavorable conditions for cell survival during storage.

In contrast, strawberry by-product extracts and diluted apple juice maintained cell viability over time, as shown in Figure 6b,c. The ability of both matrices to preserve viability of *P. frutescens* may be attributable to environmental conditions that are more favorable for cell survival than those provided by blueberry extract.

The viability of *P. frutescens* cells was further evaluated within the complete hydrogel 3 either in its proper formulation including strawberry by-product extract (see Table 1) or in a slightly modified recipe alternatively containing diluted apple juice (Figure 6e,f). No appreciable differences in cell viability were observed between the two hydrogel 3 formulations (Figure 6g). These findings, combined with the added value conferred by the presence of known antioxidants, supported the selection of hydrogel 3 containing strawberry extract as the optimal formulation, while also enabling the valorization of food-processing residues within a circular economy framework without compromising cell viability.

**Figure 7:**
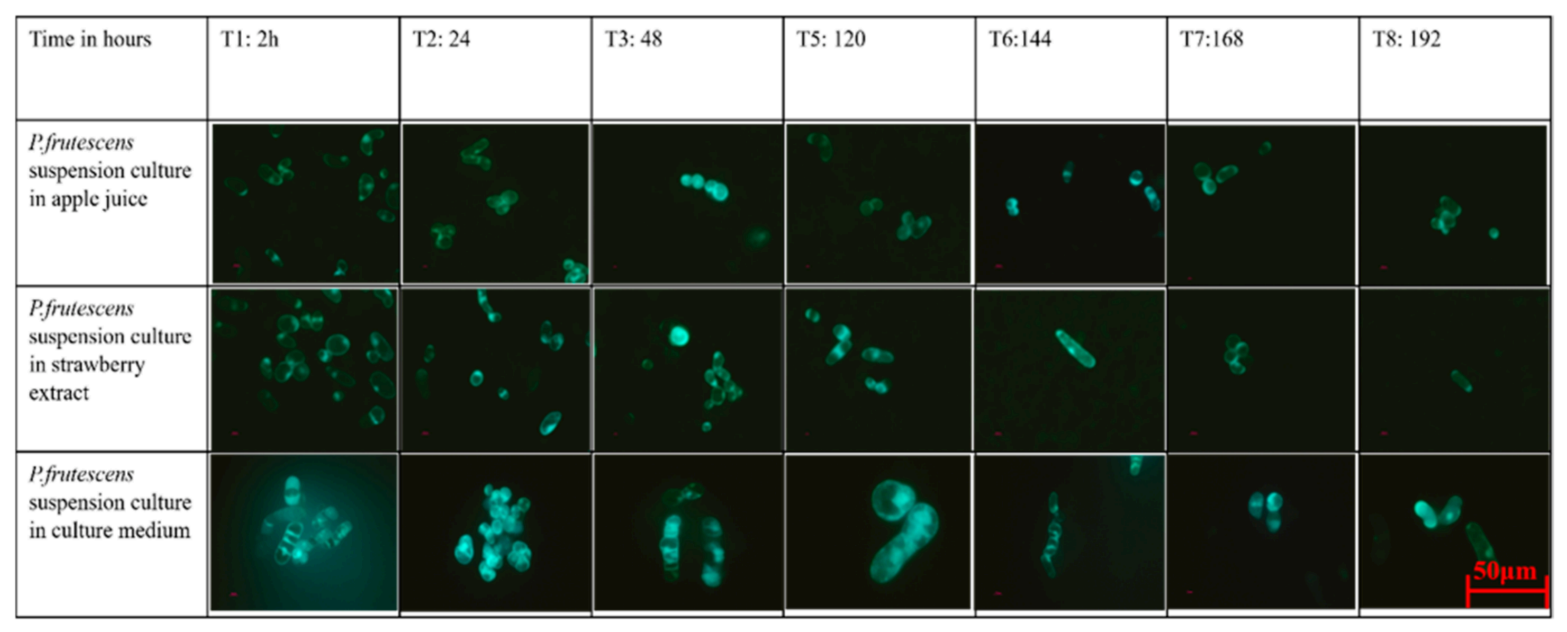
Fluorescence micrograph depicting the viability of *P. frutescens* cells resuspended in 1% strawberry by-product extract in 0.5% citric acid or clear apple juice concentrate (diluted with double-distilled water) or in its standard culture media. Images depict FDA-stained cells from T1 (2 h) to T8 (192 h, day 8). Bar = 50 µm.

In Figure 7, representative fluorescence micrographs of cells stained with FDA are shown to support these results.

### 2.4. Analytical Characterization of Hydrogels by FTIR/ATR Spectroscopy

**Figure 8:**
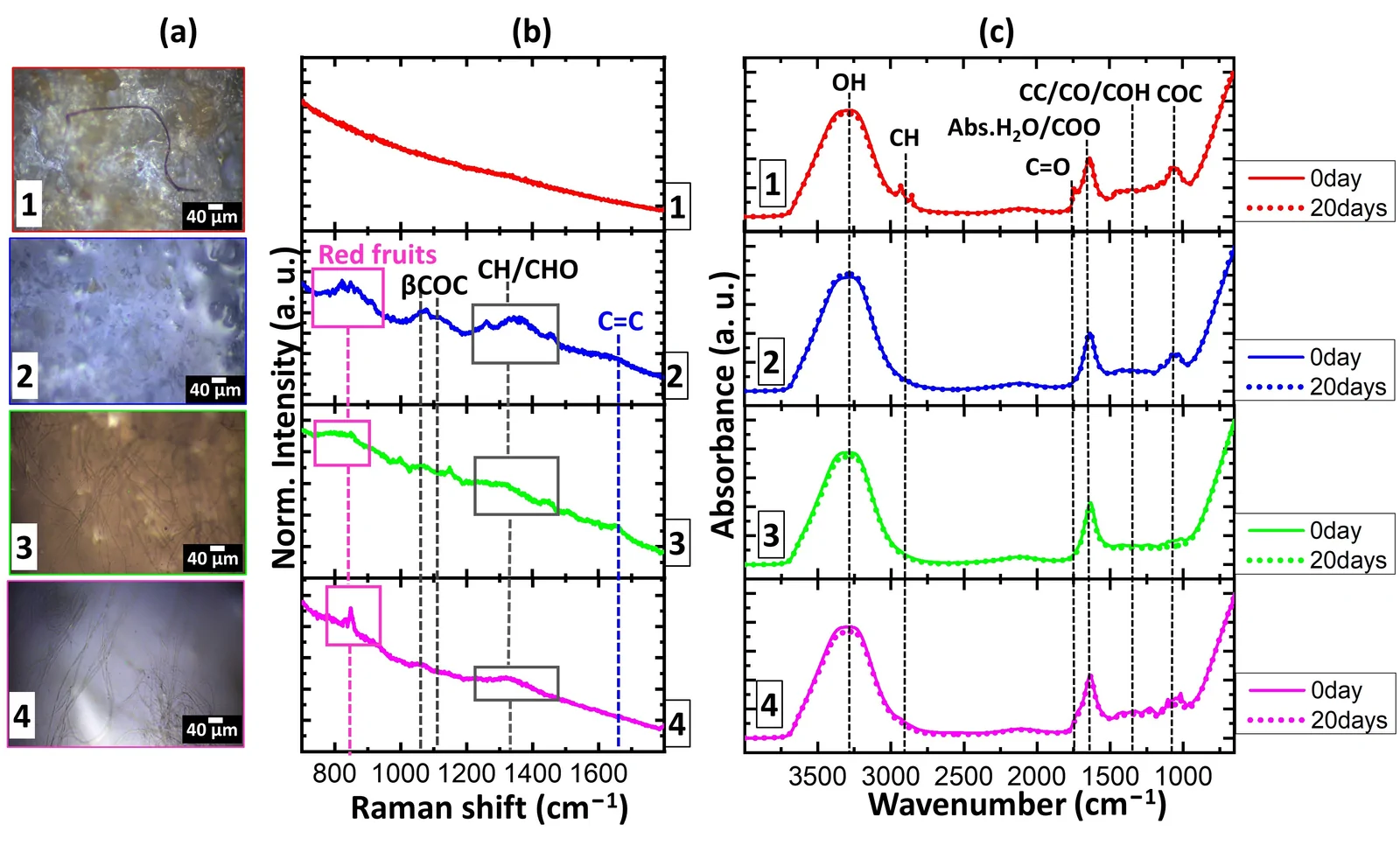
(**a**) Optical microscope photographs with 10× objective; (**b**) Raman spectra and (**c**) FTIR/ATR spectra acquired on freshly produced hydrogels (solid line) and on samples stored for 20 days at 4 °C (dashed line) along with signal attribution.

The optical microscope photographs (10× objective) and Raman and FTIR/ATR spectra of the hydrogels are reported in the following figure (Figure 8).

The microscopic analysis in Figure 8a confirms the macro-structural observations. While hydrogel 1 displays a fiber-rich morphology due to the insoluble apple fractions, hydrogel 2 exhibits a homogeneous and granular morphology that reflects the dense polysaccharide-rich matrix possessed by banana powder and locust bean gum [30,31]. On the other hand, hydrogels 3 and 4 exhibit a well-defined filamentous network. Specifically, the thin, interconnected strands visible in hydrogel 3 could be related to the calcium-pectinate scaffold, which is responsible for texture of this formulation [32]. As depicted in Figure 8b, the Raman spectral profiles of the four hydrogels exhibit variation based on the nature of the ingredients utilized. Due to the high compactness of the formulation and the presence of oily components, Raman spectra could not be successfully resolved for hydrogel 1. This limitation may possibly arise because the intrinsic autofluorescence of the ingredients completely overwhelms the naturally weak Raman scattering. Furthermore, the highly compact texture of the hydrogel 1 matrix, as highlighted by morphological analysis (Figure 8a), likely promotes local light entrapment and scattering, preventing the emitted radiation from effectively reaching the detector as clear vibrational bands. Consequently, the resulting spectrum is entirely dominated by a massive fluorescence background, making it impossible to resolve or attribute individual Raman peaks. In contrast, hydrogels 2, 3 and 4 display characteristic signals around 800 cm^−1^, attributable to red fruit extracts [33,34], with additional peaks between 1000 and 1500 cm^−1^ corresponding to βCOC and CH/COH vibrations arising from glycosidic components [35]. Moreover, a band near 1600 cm^−1^ indicates the presence of C=C double bonds. Notably, the βCOC and CH/COH glycosidic signals are marked in hydrogel 2, likely due to the abundance of polysaccharide units derived from banana powder and carob flour components.

A deeper insight into the Raman spectra of hydrogels 2 and 4 allows us to observe peaks typical of blueberry extract at 820 cm^−1^, whereas the peak at the same position in hydrogel 3 could plausibly be due to the presence of strawberry extract. On the other hand, the signal at 1600 cm^−1^ is reasonably attributable to the C=C double bonds of casein hydrolysate. The peaks’ attributions are summarized with their respective literature assignments in Table 6.

The FTIR/ATR analysis further elucidated the chemical composition of the hydrogels, as shown in Figure 8c. All spectra display a broad peak around 3200 cm^−1^, given by the overlapping of the vibrational modes of the –OH groups in absorbed water and the stretching of –OH groups in polysaccharides and pectin. The peaks near 2930 cm^−1^, associated with the vibrations of –CH groups, are clearly distinguishable only in the spectrum of hydrogel 1, while these signals are undetectable in the spectra of the other hydrogels. This finding could be attributable to the lower water content in hydrogel 1, or could be due to the presence of lignin and hemicellulose in the apple fiber, along with the hydrophobic effect of the oil. The bands at around 1750 and 1650 cm^−1^ are respectively associated with the stretching of C=O groups and with the vibrations of free carboxyl groups in carboxylate compounds. The series of peaks at wavenumbers below 1500 cm^−1^ are connected to the vibrations of the CC, CO, and COH groups, while the peaks around 1200 cm^−1^ are indicative of COC glycosidic bonds [36]. The FTIR/ATR spectra acquired on hydrogels stored for 20 days at 4 °C are perfectly superimposable on those of freshly produced food matrices (Figure 8c, dashed lines). This spectral consistency suggests that, under the specified storage conditions, the hydrogels undergo no appreciable oxidative degradation or notable compositional alterations. Consequently, these findings support the conclusion that the hydrogels maintain their physicochemical integrity over the 20-day period. It is important to clarify, however, that these observations focus exclusively on physicochemical and molecular structural constancy, as microbiological parameters were not evaluated.

**Figure 9:**
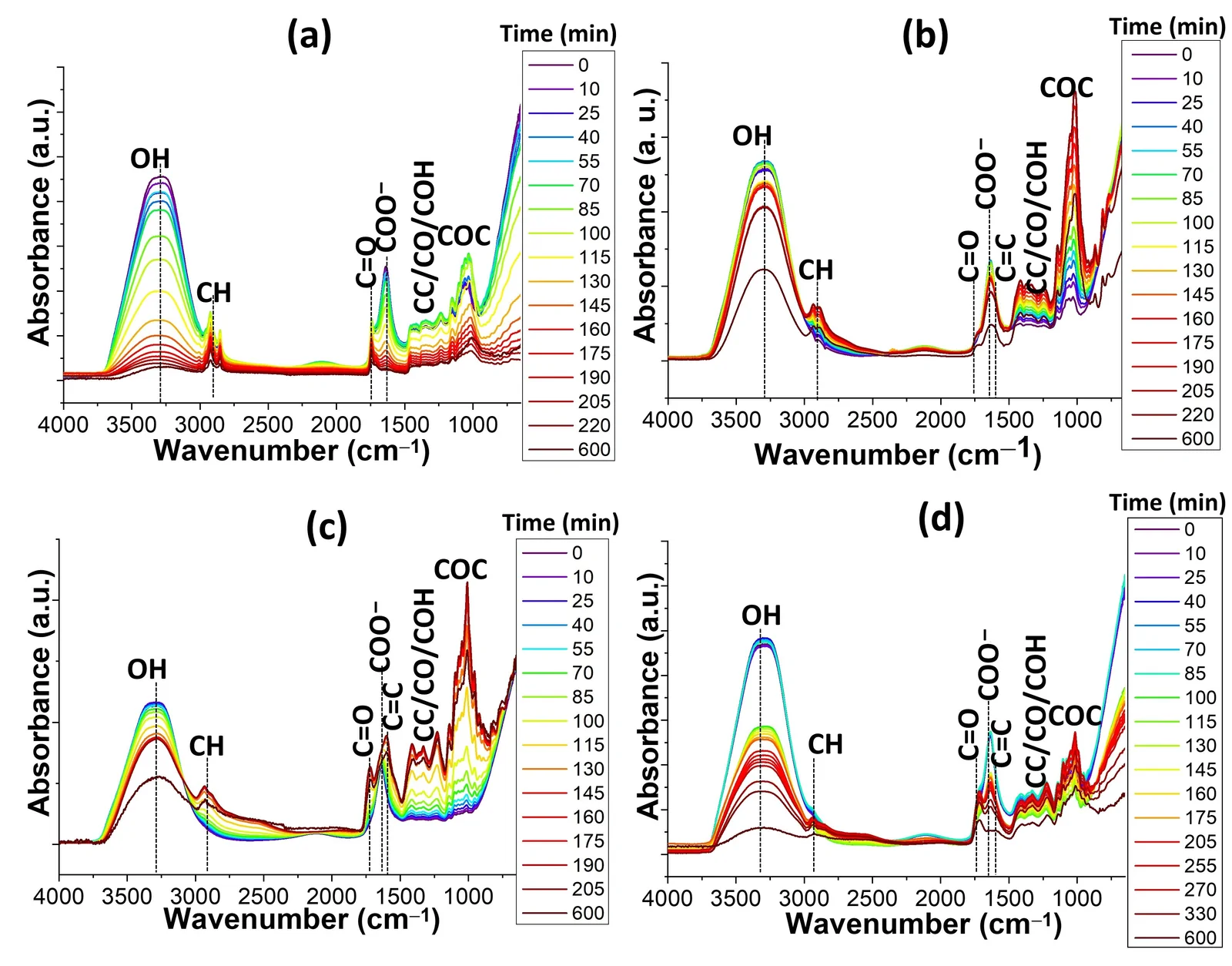
Time-lapse FTIR/ATR spectra acquired for hydrogels (**a**) 1, (**b**) 2, (**c**) 3 and (**d**) 4.

In this context, FTIR/ATR spectroscopy analysis was performed at regular intervals of time to accomplish temporal visualization of the dehydration and structural evolution of the four hydrogels over a 600 min period. In Figure 9 the time-lapse FTIR/ATR spectra are reported.

Hydrogel 1 (Figure 9a), characterized by apple fiber and oil, exhibits a rapid reduction in the OH signal within the first 175 min, likely due to the hydrophobic nature of the oil phase, which promotes water exclusion. Notably, the CH stretching peaks at approximately 2930 cm^−1^ remain sharp and well-resolved even after 600 min. This persistence could plausibly be correlated with the molecular-level stability of the aliphatic chains derived from the fiber and oil components. This spectroscopic evidence reasonably indicates that, despite the ongoing dehydration process, the hydrophobic backbone of these molecules remains structurally intact, demonstrating robust physicochemical stability within the hydrogel matrix. The observed increase in the intensity of the C=O ester groups, alongside the concomitant decrease in the COO- peak, suggests that cross-linking may occur between the polymer chains present in the hydrogel [37,38,39,40,41]. Given the structural rigidity achieved by hydrogel 1 at the end of the drying process, it is reasonable to suggest that, as water leaves the system, the carboxyl groups of the apple fiber may engage in direct hydrogen bonding or ester-like interactions. This could contribute to the final rigid consistency of the hydrogel 1 system.

A different trend is observed for hydrogel 2 (Figure 9b). While the ratio between the intensity of C=O and COO- signals remains relatively stable, the resolution of the glycosidic bond signals (C-O-C fingerprint region) increases sharply starting from 100 min. This could be interpreted as an indication that the combination of banana powder and locust bean gum leads to a highly ordered polysaccharide network. In this context, the relative stability of the C=O and COO- peaks suggests that the structural integrity of hydrogel 2 is primarily driven by the concentration of sugars and gums into a dense, saccharide-rich glass, rather than ionic shifts.

The spectral evolution of hydrogel 3 (Figure 9c) highlights a sophisticated multi-step cross-linking process driven by the presence of CaCl_2_ and casein [10,11]. A key diagnostic feature is the significant increase in the relative intensity ratio between the C=O and COO^−^ peaks as drying progresses beyond the first 100 min. This shift can tentatively be associated with the transition from free carboxylate groups to coordinated or protonated states. This trend appears compatible with the ‘egg-box’ model, wherein calcium ions may bridge the low-methoxyl pectin (LMP) chains. While the presented FTIR/ATR analysis aligns with this mechanism, it should be noted that further investigations, such as rheological analysis or comparative studies with calcium-free systems, would be required to definitively confirm this cross-linking pathway. Beyond this ionic assembly, the network could be further reinforced by the presence of casein. The protein’s Amide I and II signals overlap with the carboxylate region, creating a broader and more intense absorption band. Such a spectral feature can be tentatively explained by the potential formation of complexes involving the interaction between pectin and protein functional groups.

Simultaneously, the COC stretching vibrations become progressively more intense and well-defined over time. This sharpening of the “fingerprint” region may indicate the development of a highly ordered and rigid polysaccharide backbone, as the polymer chains are forced into proximity by the ionic bridges.

It is interesting to note that, analogously to hydrogel 2, the spectra of hydrogel 3 do not show total disappearance of the -OH stretching band even at 600 min. This persistence suggests that the hydrogel undergoes incomplete dehydration. On this basis, it is possible to argue that the ionically cross-linked network and the protein fractions may act as hygroscopic traps, retaining a fraction of “bound water” within the matrix. This internal hydration could play a crucial role, as it may potentially prevent the hydrogel from becoming brittle, even during the significant loss of free water (syneresis) suggested by the emergence of the C=C signals.

The spectroscopic profile of hydrogel 4 (Figure 9d) reveals peculiar behavior, where the chemical environment is dominated by the interaction between the pectin matrix, high concentrations of honey, and citric acid. A distinctive feature of this formulation is that the intensity between C=O and COO^−^ peaks is greater than 1 throughout the entire 600 min observation period. Considering that involvement of citric acid produces acidic conditions, the carboxyl groups are expected to remain largely protonated (-COOH) rather than ionized (-COO^−^). This occurrence may plausibly suppress the formation of ionic bridges, favoring a network based on inter-chain hydrogen bonding. Similarly to hydrogel 3, the emergence of the C=C signal during the later stages (t > 200 min) could suggest potential β-elimination mechanisms occurring within the pectin chains under acidic conditions. Based on other studies [42], it is possible to speculate that these degradation pathways might be favored in such acidic environments over time.

### 2.5. Hygroscopic Behavior of Hydrogels over Time

**Figure 10:**
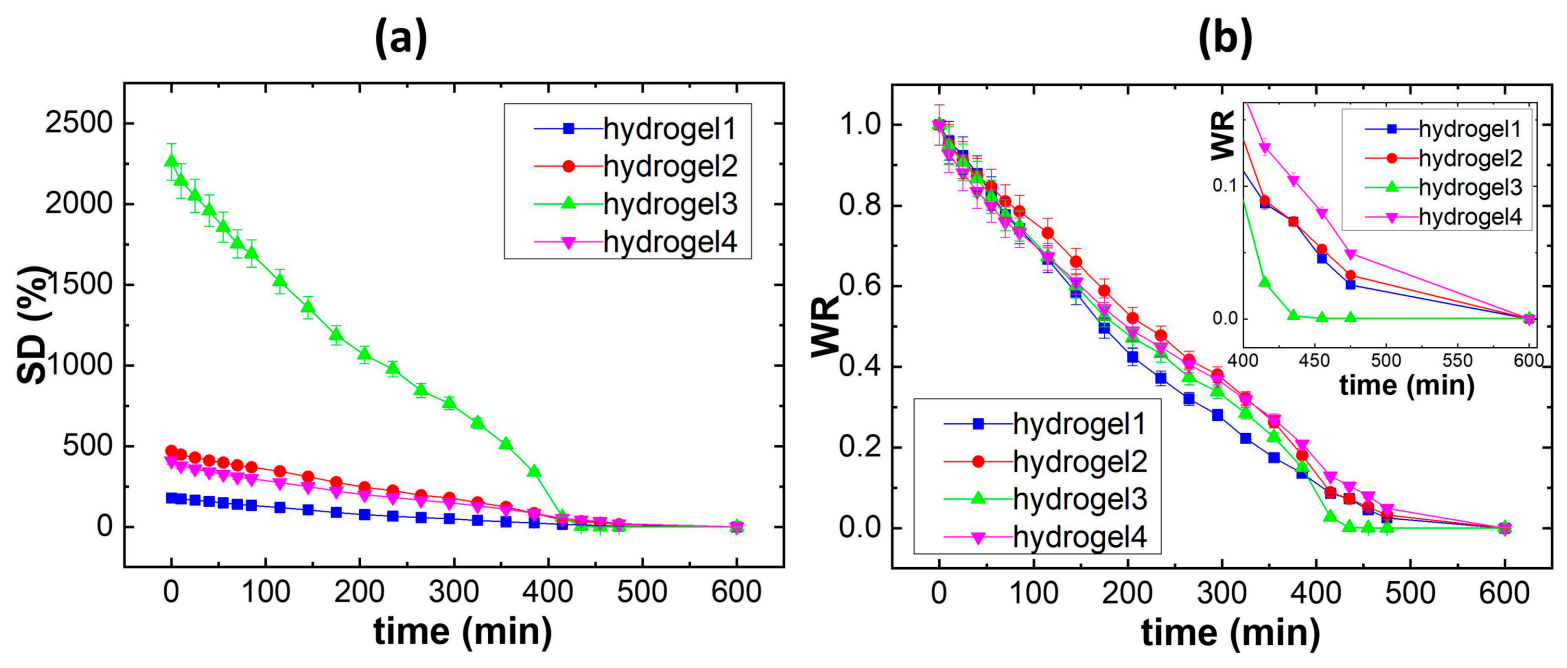
Trends of (**a**) swelling degree (SD%) and (**b**) water retention (WR) as a function of drying time. The error was calculated as the standard deviation on three sets of experiments.

To provide more insights into the hygroscopic behavior of the produced hydrogel samples, Figure 10 reports the trends of swelling degree (SD%) and water retention ratio (WR) over time.

As illustrated in Figure 10a, the SD% values measured at time zero (t = 0) are approximately 190, 500, 2200 and 480% for hydrogels 1, 2, 3, and 4, respectively. Corroborating the FTIR/ATR results, the synergistic interaction between water molecules, any ionic additives and organic molecules, such as pectin, polysaccharides, and peptides, modulates the hygroscopic behavior of the hydrogels, depending on their specific chemical composition [43,44,45]. In particular, the variation in the amount of water absorbed is likely attributed to the distinct chemical composition of the hydrogel mixtures. In perfect agreement with the FTIR/ATR spectroscopy analysis, the presence of hydrophobic substances such as oil, lignin and cellulose produces the lowest swelling capacity for hydrogel 1, which retains the least amount of water. In contrast, hydrogel 2 and hydrogel 4, which have a high content of sugary substances, show similar swelling percentages, resulting in comparable SD% values. Hydrogel 3, with its significantly higher degree of swelling, can be plausibly explained by the presence of calcium chloride ions. It could be speculated that the localized charges on these ionic species potentially enhance water retention. This rationale is supported by the assumption that these charges are likely to increase the number of polar anchoring sites, allowing for a more extensive network of hydrogen bonds with water molecules throughout the three-dimensional hydrogel structure.

However, it is worth noting that, independently of the swelling degree measured at time zero, all four hydrogels reach dehydration after approximately 400 min, revealing a notable capacity to retain water molecules over time. Among the tested formulations, hydrogels 1, 2, and 4 exhibited gradual dehydration with comparable WR trends (inset of Figure 10b). In contrast, hydrogel 3 showed a drop in water retention between 400 and 450 min. This divergence can plausibly be attributed to its specific chemical composition: as dehydration proceeds, calcium chloride concentration increases within the hydrogel network. Acting as a cross-linker, the calcium ions promote network stiffening and compaction, ultimately triggering the rapid expulsion of residual water. This interpretation is consistent with the well-established ‘egg-box’ model, which describes calcium-induced gelation in pectin systems. Although our study lacks specific calcium-binding assays or comparative analyses with calcium-free controls, these spectroscopic changes strongly suggest a similar mechanism, providing indirect evidence of such a cross-linking process occurring within the hydrogel 3 matrix.

### 2.6. Analysis of Component Interactions and Possible Gelling Mechanisms

Based on the overall results, the integration of the pH measurements with spectroscopic and hygroscopic data allows for a comprehensive overview of the structural evolution within these hydrogels.

The hydrogel 1 formulation was designed to mimic the color and texture of cookie dough, while maintaining a balanced nutritional profile. Specifically, the synergistic interaction between the glycosidic units in honey, the polymers from apple fiber, and oil molecules realistically results in a compact and dense matrix. Here, the spectroscopic and hygroscopic data suggest that water molecules are retained within the polymer network through hydrogen bonding and electrostatic interactions with polar groups. In contrast, the results obtained for hydrogel 2 likely indicate that the gelation of this matrix is primarily driven by the reorganization of pectin chains. In this aqueous environment, it is possible to hypothesize that the presence of amidated pectin, citric acid, and sugars from locust bean gum and banana powder may contribute to the formation of a three-dimensional network, potentially maintaining the molecular arrangement, even in the absence of exogenous ionic cross-linkers. This interpretation is based on the inherent chemical interactions expected between these biopolymers and small molecules, although further investigation would be required to fully elucidate the specific contribution of each component to this stabilization. A different scenario is observed for hydrogel 3, where the gelation process could be plausibly associated with calcium–pectin cross-linking. While evidence of calcium binding is not provided directly, the combination of spectral changes and hygroscopic data is consistent with mechanisms reported in the scientific literature for similar pectin-based systems. On this basis, it is possible to interpret the obtained findings as an indication that calcium ions promote the formation of a cross-linked network, effectively bridging the pectin chains. As well-documented in the literature, calcium chloride releases Ca^2+^ ions that form ionic bonds with the carboxyl groups of pectin, organizing the polysaccharide chains into a stable, structured network [7,10,11,12,13]. In this context, it can be inferred that the non-amidated pectin (PRIME 521), selected for the formulation of hydrogel 3, acts as the primary driver for the formation of the ionically cross-linked network. Its high reactivity toward Ca^2+^ ions could plausibly facilitate the development of an ‘egg-box’ assembly, which is fundamental to the structural stabilization of the matrix.

Given such considerations, together with the UV-Vis spectroscopic data and pH measurements, it can be hypothesized that the role of the ingredients is not merely physical. Specifically, it is plausible that citric acid, by modulating the pH, creates a favorable chemical environment, while the casein and sugars from the strawberry extract may actively contribute to the cohesion and viscosity of the matrix. Based on these findings, it is reasonable to interpret the observed behavior as the result of a synergistic interaction between the molecular species present, which likely promotes the structural stability of the final system. Regarding hydrogel 4, it can be hypothesized that the network formation arises from the interplay between fir honey, bilberry extract, and pectin. Based on the chemical components involved, it is plausible that hydrogen bonds form not only between pectin and water but also with the glycosidic units present in the extracts. Furthermore, the role of citric acid in modulating the pH to favor gelation, combined with the contribution of fir honey to the matrix viscosity, may explain the resulting pasty and stable consistency of the hydrogel.

Collectively, these findings demonstrate that while the final consistency is a common macro-structural goal, it is achieved through distinct chemical pathways, ranging from ionic bridging to acid-induced hydrogen bonding. All these molecular rearrangements are clearly traceable through the temporal shifts in their spectroscopic signatures. While these interactions represent a reasonable interpretation of the system’s behavior, it should be noted that they are inferred from formulation and macroscopic observations, and further investigations would be required to fully elucidate the specific molecular mechanism behind this physicochemical stability.

### 2.7. Effect of the Presence of Plant Cells on the Hygroscopic Behavior of Hydrogel 3

**Figure 11:**
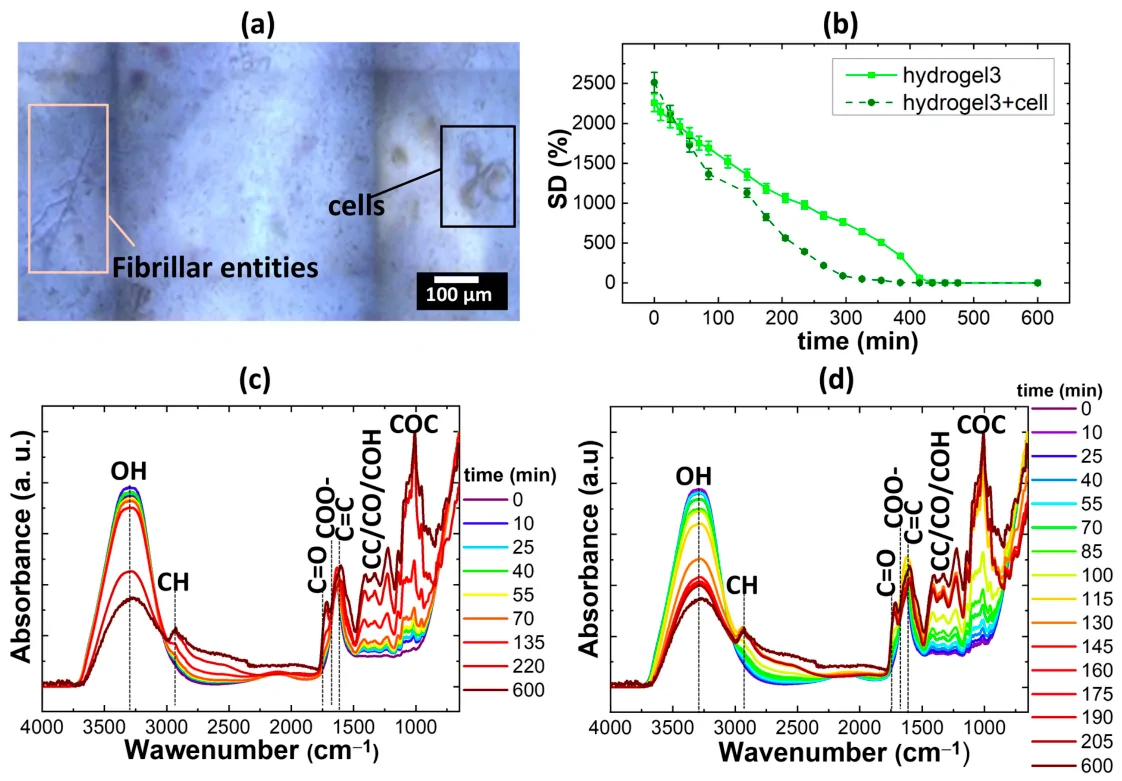
(**a**) optical microscope photo of hydrogel 3 with cells, (**b**) trend of swelling degree over time for hydrogel 3 with and without cells, and FTIR/ATR over time for hydrogel 3 (**c**) with and (**d**) without cells.

To evaluate the potential of the developed matrices as bioactive scaffolds, the following section examines how the embedding of living plant cells influences the hygroscopic behavior, water dynamics, and physicochemical integrity of the molecular arrangement in the hydrogel matrices. Hydrogel 3 was selected as the most suitable candidate for cell embedding, primarily due to its high swelling capacity. This hydrogel appears to feature a stable network, which, based on indirect spectroscopic evidence, may be consistent with an ‘egg-box’ cross-linking mechanism. Such a structure could provide a highly hydrated and mechanically resilient environment, potentially mimicking the natural extracellular matrix of plant tissues more effectively than the other tested formulations. Figure 11 shows the optical microscope photo of hydrogel 3 with cells, the trend of swelling degree over time for hydrogel 3 with and without cells, and the time-lapse FTIR/ATR spectra for hydrogel 3 with and without cells.

The microscopic characterization in Figure 11a provides direct visual evidence of the successful integration of biological components within the synthetic matrix. Specifically, the image reveals the presence of roundish-shaped cells effectively embedded within the hydrogel network. This finding supports the consideration that the cells are well-integrated and stable within the produced matrix, suggesting a biocompatible environment that maintains the integrity of the cellular structures alongside visible fibrillar entities.

The study of the dehydration kinetics, reported as swelling degree (SD%) as a function of time in Figure 11b, highlights how the presence of biological matter influences the material’s interaction with water. Regardless of the presence of plant cells, the initial SD% is comparable for both samples, reaching values around 2500%. This indicates that the inclusion of cells does not significantly alter the primary capacity of the pectin-based matrix to absorb water at time zero. On the other hand, the embedding of cells within the hydrogel matrix clearly modulates the water retention behavior of the final material. Specifically, the hydrogel containing cells reaches an SD% approaching zero after 300 min of drying, whereas the reference hydrogel (without cells) maintains moisture for longer, reaching the same state only after 400 min. The presence of living plant cells within the hydrogel seems to play a key role in modulating these water dynamics. These cells remain turgid due to osmotic pressure, driven by water and solute content in the cytoplasm and vacuole, both of which are critical for sustaining metabolic activity and membrane exchange. Their ability to absorb or release water in response to environmental stimuli directly influences the hydrogel’s hydration status. Moreover, plant cells can physically interact with the pectin matrix and Ca^2+^ ions, derived from CaCl_2_ dissociation within the hydrogel matrix. These interactions may serve as anchoring points within the matrix, contributing to the physicochemical and molecular stabilization of the three-dimensional network. Such cell–matrix interactions enhance the hydrogel’s structural integrity and modulate its water-holding capacity.

Despite the differing hygroscopic properties observed in the swelling tests, the comparison of FTIR/ATR spectra for the hydrogel with (Figure 11c) and without cells (Figure 11d) suggests that the underlying dehydration mechanisms remain fundamentally unchanged. In the first 100 min of the process, a decrease in the ratio between the intensity of the C=O and COO^−^ peaks is observed. This corroborates the hypothesis based on a mechanism initially dominated by cross-linking, where the carbonyl groups (C=O) participate in esterification reactions to form COO bonds, stabilizing the scaffold. The emergence of signals attributable to C=C stretching in the IR spectra appears to be consistent with β-elimination mechanisms. Based on these spectroscopic findings, it is possible to interpret this pathway as a significant factor in the structural evolution of the gel network. While this interpretation aligns with established chemical mechanisms for pectin dehydration, it should be noted that these signals provide indirect evidence; thus, the occurrence of β-elimination is proposed as a likely contributing mechanism in the development of the gel architecture [45]. This chemical transition is clearly visible in both systems, indicating that the presence of embedded plant cells does not significatively interfere with the primary chemical pathways of pectin–calcium network formation.

Overall, the inclusion of plant cells appears to improve the predictability and performance of the hydrogel matrix, particularly in hydrogel 3, by stabilizing water retention. This characteristic is especially relevant for its potential application in functional food products. Based on the observed dehydration kinetics and swelling profiles, it is possible to hypothesize that the embedded living plant cells do not merely act as passive nutritional ingredients. Instead, our data suggest they might function as active components that could help modulate internal moisture migration. This biological presence may tentatively contribute to the overall physicochemical integrity of the system, potentially offering a sustainable way to mitigate syneresis tendencies over time without relying on synthetic additives. Furthermore, this integration presents a promising dual perspective. On one hand, vital cells appear to influence the water-retention behavior of the formulation through potential water-binding pathways. On the other hand, they could concurrently serve as a protected, biocompatible vehicle for the delivery of plant-derived bioactive compounds. Importantly, under the tested storage conditions (i.e., exposure to air at 4 °C), the FTIR and pH data showed no evidence of oxidative degradation or significant compositional shifts, which confirms the chemical and physical stability of these food-grade hydrogels over time. It should be noted, however, that while these results demonstrate physicochemical stability, this study did not assess microbiological parameters. Therefore, our conclusions regarding the ‘stability’ of the hydrogels are limited to their chemical and structural integrity.

## 3. Conclusions

In this study, food-grade hydrogels integrating low-methoxyl pectins, diverse agro-industrial by-products, and living *P. frutescens* plant cells were developed and characterized by coupling a series of spectroscopic, hygroscopic, and physicochemical analyses. The chemical formulation of the four hydrogels was tuned based on the structural properties of the gelling agents and the nature of the by-products. Specifically, non-amidated pectin potentially promoted hydrophobic fiber–oil interactions in hydrogel 1 and calcium-mediated cell encapsulation in hydrogel 3. Conversely, amidated pectin in hydrogels 2 and 4 (blended with locust bean gum and honey, respectively) likely favored dense, associative hydrogen-bonded configurations. Long-term monitoring at 4 °C demonstrated robust physicochemical integrity regarding pH and moisture content across all formulations, alongside the effective preservation of embedded bioactive polyphenolic profiles, as disclosed by UV-Vis spectroscopy analysis.

The integration of micro-Raman and time-resolved FTIR-ATR spectroscopies suggested internal molecular rearrangements. In particular, the interplay between the components involved in the chemical formulation of the hydrogels appears to modulate dehydration behaviors. These pathways range from the rapid, hydrophobic-driven water exclusion observed in hydrogel 1 to the high water-binding capacity of hydrogel 2, and from the inferred calcium-mediated ‘egg-box’ routes in hydrogel 3 to the protonated, sugar-associated configuration of hydrogel 4.

Crucially, the scientific novelty of this work moves beyond the mere utilization or upcycling of agro-industrial by-products. Instead, the core innovation lies in the comprehensive, multi-parametric rationalization of the hydrogels’ properties achieved by systematically combining time-resolved spectroscopic, kinetic, and hydrodynamic techniques. Through this integrated framework, the present study elucidates how the incorporation of living plant cells is not merely passive. Conversely, the living cells actively modulate the hydrogel’s dehydration kinetics and swelling capacity, and the temporal evolution of the chemical composition over time, while maintaining cellular viability. The ability of incorporated plant cells to modulate water retention can be particularly relevant for food applications, as the ability to retain water is closely associated with physicochemical integrity, prevention of syneresis, and maintenance of product quality during storage. Therefore, the presence of vital plant cells can contribute not only to the potential supply of plant-derived bioactive compounds but also to the physical robustness of the hydrogel.

Within the framework of plant cell agriculture, the developed living hydrogels may represent a promising conceptual platform for the integration and stabilization of plant cells within structured food matrices, where biological components can actively contribute to the physicochemical properties of the system. Considering that the experiments were conducted in duplicate, the statistical analyses were intended for exploratory purposes, and the observed trends warrant confirmation through future studies with greater experimental replication. Further statistical analysis investigations will be required to evaluate the scalability, nutritional potential, and processing performance of such systems. To translate these chemical and hygroscopic insights into advanced manufacturing applications, upcoming studies will be strictly dedicated to a comprehensive evaluation of extrusion performance and mechanical deposition parameters to definitively validate these sustainable formulations as functional inks for extrusion-based 3D food printing.

## 4. Materials and Methods

### Hydrogel Ingredients and Recipes

The gelling agents used in this study were two types of commercial low-methoxyl (LM) pectin. PRIME 521 (Danisco, Copenhagen, Denmark) is a non-amidated citrus peel pectin characterized by a degree of esterification (DE) of 35% and a pH range of 4.0–4.8 (1% aqueous solution). It was selected for its high calcium reactivity, which facilitates the formation of ionically cross-linked networks. Pectin Amid AF 005 (Herbstreith & Fox GmbH & Co. KG, Neuenbürg, Germany) is an amidated LM pectin with a DE of 30–38% and a degree of amidation (DA) of 7–14%, standardized with calcium citrate.

To develop hydrogel suitable for incorporating plant cells and/or high-value molecules derived from agri-food by-products, several preliminary formulation tests were conducted. These activities led to the design and definition of four final formulations (hydrogel 1–hydrogel 4), based on plant extracts and natural biopolymers. One of the formulations (hydrogel 3) was also evaluated in both the absence and presence of *Perilla frutescens* cells, used as a plant model.

The four final formulations of the developed hydrogels are reported below:

- Hydrogel 1: 1% *w*:*v* blueberry by-product (Rigoni di Asiago Srl, Asiago–Italy) extract in 5% *w*:*v* citric acid (66.67% *v*:*v*), SD apple fiber (22.92% *w*:*v*; Rigoni di Asiago Srl, Asiago–Italy), high-oleic oil (1.60% *w*:*v*; Rigoni di Asiago Srl, Asiago–Italy), and flower honey (8.01% *w*:*v*; Rigoni di Asiago Srl, Asiago–Italy).
- Hydrogel 2: 1% *w*:*v* blueberry by-product in 0.5% *w*:*v* citric acid (14.95% *v*:*v*), amidated pectin in diluted apple juice (74.74% *w*:*v*), banana powder (2.84% *w*:*v*), and locust bean gum (7.47% *w*:*v*).
- Hydrogel 3: designed for the inclusion of plant cells in a jelly-like structure, with properties suitable for their preservation with 1% *w*/*v* strawberry by-product extract in 0.5% *w*:*v* citric acid, low-methoxyl, non-amidated pectins (LMPs) (3% *w*:*v*), casein hydrolysate (0.50% *w*:*v*), and CaCl_2_ 80 mM (final concentration of CaCl_2_ = 11.25 mM; 14% *v*:*v*).
- Hydrogel 4: 1% *w*:*v* blueberry by-product extract in 5% *w*:*v* citric acid (85% *v*:*v*), low-methoxyl, non-amidated pectins (LMPs) (10% *w*:*v*) and fir honey (5% *w*:*v*; Rigoni di Asiago Srl, Asiago–Italy).

*Embedding of cells into hydrogel 3*

Hydrogel 3 was specifically intended to embed plant cells. The present section describes the methods for the preparation of solutions aimed at resuspending cells. *P. frutescens* suspension culture was sieved through a sterile stainless steel sieve (<100 µm) to select a cell fraction of suitable size to pass through the different nozzles used for deposition. This fraction was then centrifuged at 950 *g* to remove the culture medium. Total cell count was determined using the Thoma chamber (E.Hartnack, Berlin-Steglitz, Berlin, Germany) under an inverted microscope, while cell viability was quantified by the FDA test.

The compatibility of by-product extracts (strawberry and blueberry) or apple juice for cell resuspension was analyzed as a function of the preservation of cell viability at 4 °C. Cells (100 µm-sieved 1 × 10^6^ cells/mL) were resuspended in:

- Strawberry and blueberry by-product extracts (1% *w*:*v* in 0.5% *w*:*v* citric acid);
- Clear apple juice concentrate (diluted to 4.91% *w*/*v* in double-distilled water).

As a control, cells were resuspended in their standard culture medium (MS with 2,4-D 0.5 mg/L and BAP 1 mg/L) and cell viability was assessed in all the resuspension solutions by the FDA test (100 µL of cell suspension was mixed with 0.1 µL of FDA and incubated for 5 min, then diluted 1:8 in the respective solutions). Cell viability was monitored at different time points (2 h, 24 h, 48 h, 120 h, 144 h, 168 h, 192 h) and the experiment was performed in duplicate.

Cell viability was assessed in the complete hydrogel. Combinations of cells and recipe ingredients were as follows:

- *P. frutescens* cell suspension (1 × 10^6^ cells/mL in culture media) in 1% *w*/*v* strawberry-processing by-product extracts in 0.5% *w*/*v* citric acid and low-methoxyl, non-amidated pectin (LMP) + casein hydrolysate + CaCl_2_.
- *P. frutescens* cell suspension (1 × 10^6^ cells/mL in culture media) in clear diluted apple juice concentrate and low-methoxyl, non-amidated pectin (LMP) + casein hydrolysate + CaCl_2_.

Viability was determined by incubating 250 mg of samples A–D with 500 µL of Milli-Q water and 1.5 µL of FDA in the dark for 5 min. Evaluation of viability was performed at different time points (T1: 2 h, T2: 24 h, T3: 48 h, T4: 72 h).

Viability was determined by fluorescence microscopy as the percentage of FDA-positive cells over the total number of observed cells. Multiple independent microscopic fields were analyzed for each sample to calculate viable values. This procedure was applied to all experimental conditions, including both resuspension solution and hydrogel formulation.

*pH and moisture stability*

Stability assessments of the hydrogels were conducted, focusing on pH stability and changes in total moisture content (0 to 5 days). The total moisture of the samples and its variation over time were measured using a MA 50X2ICAWH Moisture Analyzer (Radwag, Radom, Poland). For monitoring pH variations over time (0–96 h), a pH meter (HANNA instrument, pH/ORP, and ORP testers HI70022) was employed. All experiments were performed in duplicate. Statistical analysis (one-way ANOVA followed by Tukey’s post hoc test) was performed for exploratory purposes.

*UV-vis spectrum*

The UV-Vis spectra of the hydroalcoholic extracts (MeOH: H_2_O (70:30 *v*/*v*)) obtained from 150 mg of the complete formulation were compared with those from the single ingredients (30 mg).

*Raman and FTIR/ATR spectroscopy analyses*

Raman spectra were acquired in the range between 700 and 1700 cm^−1^ by a Horiba XploRA Plus system (HORIBA ITALIA Srl, Rome, Italy) equipped with a 785 nm laser source for excitation. The laser power was set at 50 mW, exposure time at 10 s, and diffraction grating at 1200 grooves per millimeter along with a 10 X objective lens for magnification. Origin 2018 software was employed to subtract the baseline and to normalize the spectra.

Fourier Transform Infrared Spectroscopy (FTIR) with an Attenuated Total Reflectance (ATR) setup was performed using a Perkin-Elmer Spectrum 100 FT-IR instrument (supplied by PerkinElmer Scientifica Italia S.r.l., Milano, Italy). The spectrometer was outfitted with a Horizontal ATR (HATR) accessory, which included a zinc selenide (ZnSe) prism. Spectral data acquisition ranged from 600 to 3500 cm^−1^ by performing 8 scans. The tested materials were situated in direct contact with the surface of the ZnSe crystal. Using FTIR/ATR analysis, changes in the chemical composition of the hydrogels over time were studied: hydrogel samples (approximately 600 mg in weight and 2 × 1 × 0.5 cm^3^ in volume) were analyzed at regular intervals to examine the functional groups interacting with water within the hydrogels.

FTIR analysis was also used to conduct morphological and compositional studies on hydrogels that had been stored for 20 days in the refrigerator at +4 °C. These tests were performed through optical microscope photographs of the samples stored for 20 days at 4 °C, and by comparing the FTIR/ATR spectra acquired from freshly produced hydrogels and those stored for 20 days at 4 °C.

*Deswelling behavior and water release over time*

The water release behavior of the produced materials was studied over time, depending on the different hydrogel compositions and the various hydrogel formation mechanisms. Specifically, the swelling degree (SD%) and water retention (WR%) values over time were calculated using Equations (1) and (2) [46]:

(1) $$SD=\;\frac{M_{t}-\;M_{d}}{M_{d}}$$

(2) $$WR=\frac{M_{t}-M_{d}}{M_{s}-M_{d}}$$

where $M_{d}$ is the weight of the hydrogel when no further weight changes are recorded after incubation at ambient temperature and humidity (dehydrated hydrogel); $M_{s}$  is the weight of the swollen hydrogel at the time point t = 0; and $M_{t}$  is the sample’s weight at the testing time (t).

All data are related to the drying process at a temperature of 25 °C and ambient humidity.

The hygroscopic behavior of hydrogel 3 in the presence of cells (*Perilla frutescens*) was evaluated, studying the effects through morphological, structural analyses, and weight loss measurements. These analyses were performed using optical microscope photographs, FTIR/ATR spectra, and the swelling degree over time of hydrogel 3, both with and without cells.

## Figures and Tables

**Table 1:** Composition of the four hydrogel recipes.

Hydrogel	Ingredients
1	Spray-dried blueberry by-products (1 wt%)
	Citric acid (5 wt%)
	Spray-dried apple fiber (22.92 wt%)
	High-oleic oil (1.60 wt%)
	Flower honey (8.01 wt%)
2	Spray-dried blueberry by-products (1 wt%)
	Citric acid (0.5 wt%)
	Amidated pectin in diluted apple juice (74.74 wt%)
	Banana powder (2.84 wt%)
	Locust bean gum (7.47 wt%)
3	Spray-dried strawberry by-products (1 wt%)
	Citric acid (0.5 wt%)
	Low-methoxyl, non-amidated pectins (LMPs) (3 wt%)
	Casein hydrolysate (0.50 wt%)
	CaCl_2_ 11.25 mM (reported as molarity)
4	Spray-dried blueberry by-products (1 wt%)
	Citric acid (5 wt%)
	Low-methoxyl, non-amidated pectins (LMPs) (10 wt%)
	Fir honey (5 wt%)

**Table 2:** Data obtained by the analysis of UV-Vis spectra of hydrogel 1.

ƛ (nm)	Putative Class of Compounds	Hydrogel 1	SD Apple Fiber	SD Blueberry By-Products	Honey
282	Flavonoids	3967	2511	0.619	0.405 (293 nm)

**Table 3:** Data obtained by the analysis of UV-Vis spectra of hydrogel 2.

ƛ (nm)	Putative Class of Compounds	Hydrogel 2	Banana Powder	Amidated Pectin	Locust Bean Gum	Diluted Apple Juice	SD Blueberry By-Products
265	Phenolic acids	0.656	0.464	-	0.384	-	
280	Flavonoids	-	-	-	-	0.123	0.123

**Table 4:** Data obtained by the analysis of UV-Vis spectra of hydrogel 3.

ƛ (nm)	Putative Class of Compounds	Hydrogel 3	Pectin	Casein Hydrolysate	SD Strawberry
280	Flavonoids Amino acids	0.382	-	0.317	0.634
320	Flavonoids	0.257	-	-	-
370	Flavonols	0.027	-	-	0.118

**Table 5:** Data obtained by the analysis of UV–visible spectra of hydrogel 4.

ƛ (nm)	Putative Class of Compounds	Hydrogel	Pectin (LMP)	Honey (Fir)	SD Blueberry
282	Flavonoids	1.772	0.180	0.238	0.927

**Table 6:** Raman band assignments and spectral attributions for the developed hydrogels.

Signal Position (cm^−1^)	Vibrational Assignment	Hydrogel Occurrence	Associated Component/Ingredient Source	References
800–820	Aromatic ring breathing/skeletal vibrations	Hydrogels 2, 3, 4	Anthocyanins and phenolics from red fruit extracts (blueberry in H2/H4; strawberry in H3)	[31,32]
1000–1500	β-(C-O-C) glycosidic bond stretching	Hydrogels 2, 3, 4 (most prominent in H2)	COC glycosidic linkages in polysaccharide units	[33]
1200–1500	C-H/C-O-H deformations	Hydrogels 2, 3, 4 (most prominent in H2)	Polysaccharide backbone	[33]
~1600	C=C ethylenic/aromatic ring stretching	Clearly detectable in hydrogel 3	Conjugated double bonds in polyphenolic extracts and protein fractions	[31,33]

## Data Availability

The data that support the findings of this study are available from the corresponding author upon reasonable request.
